# Modulation of intracortical inhibition during physically performed and mentally simulated balance tasks

**DOI:** 10.1007/s00421-020-04577-1

**Published:** 2021-02-19

**Authors:** A. Mouthon, J. Ruffieux, W. Taube

**Affiliations:** Faculty of Science and Medicine, Medicine Section, Department of Neurosciences and Movement Sciences, Movement and Sport Sciences, University of Fribourg, Fribourg, Switzerland

**Keywords:** Action observation, Balance control, Mental simulation, Postural task, SICI, TMS

## Abstract

**Purpose:**

Action observation (AO) during motor imagery (MI), so-called AO + MI, has been proposed as a new form of non-physical training, but the neural mechanisms involved remains largely unknown. Therefore, this study aimed to explore whether there were similarities in the modulation of short-interval intracortical inhibition (SICI) during execution and mental simulation of postural tasks, and if there was a difference in modulation of SICI between AO + MI and AO alone.

**Method:**

21 young adults (mean ± SD = 24 ± 6.3 years) were asked to either passively observe (AO) or imagine while observing (AO + MI) or physically perform a stable and an unstable standing task, while motor evoked potentials and SICI were assessed in the soleus muscle.

**Result:**

SICI results showed a modulation by condition (*F*_2,40_ = 6.42, *p* = 0.009) with less SICI in the execution condition compared to the AO + MI (*p* = 0.009) and AO (*p* = 0.002) condition. Moreover, switching from the stable to the unstable stance condition reduced significantly SICI (*F*_1,20_ = 8.34, *p* = 0.009) during both, physically performed (− 38.5%; *p* = 0.03) and mentally simulated balance (− 10%, *p* < 0.001, AO + MI and AO taken together).

**Conclusion:**

The data demonstrate that SICI is reduced when switching from a stable to a more unstable standing task during both real task execution and mental simulation. Therefore, our results strengthen and further support the existence of similarities between executed and mentally simulated actions by showing that not only corticospinal excitability is similarly modulated but also SICI. This proposes that the activity of the inhibitory cortical network during mental simulation of balance tasks resembles the one during physical postural task execution.

## Introduction

Motor imagery (MI) and action observation (AO) are two forms of mental simulation that have been shown to be efficient in improving motor learning and rehabilitation (Buccino 2014; Mulder 2007; Ste-Marie et al. 2012). Indeed, in 2001 Jeannerod postulated the existence of a similar neural system between execution and mental simulation of tasks—the so-called Simulation Theory—that may explain why MI and AO practice improve motor skills (Jeannerod 1995, 2001). Recently, motor imagery during action observation (AO + MI) has been proposed as another alternative (for overview see Eaves et al. 2016b; Vogt et al. 2013), and this combination seems to improve physical performance even more efficiently than MI or AO alone (Bek et al. 2016; Smith and Holmes 2004; Wright and Smith 2009). However, studies comparing directly neural mechanisms involved in execution and AO + MI are missing. Indeed, previous studies on AO + MI compared it with MI and/or AO (Eaves et al. 2016a; Ohno et al. 2011; Sakamoto et al. 2009; Tsukazaki et al. 2012; Wright et al. 2014, 2016,), but not with a real execution of the imagined task. Moreover, those studies were all performed on mental simulation of upper limb movements, while studies on the neural mechanisms of mental simulation of lower limb movements are missing.

From studies investigating the physical execution of postural tasks it is known that the primary motor cortex (M1) plays an important role in ensuring upright balance and displays increased excitability with increasing task difficulty (M1; Beloozerova et al. 2003; Taube et al. 2006, 2008; Tokuno et al. 2009). In addition, increases in postural task difficulty were shown to be associated with decreases in intracortical inhibition (Mouthon and Taube 2019; Papegaaij et al. 2014, 2016a, b; Soto et al. 2006). Thus, there is a consistent picture for M1 of increased excitability and reduced intracortical inhibition when switching from simple to more challenging postural tasks. However, so far, no study has compared task-specific changes in neural mechanism implicated during execution of physical postural task and during AO + MI of the same tasks.

Moreover, regarding neural mechanisms involved in mental simulation, there is a large body of literature for MI and AO alone (for overview see Fadiga et al. 2005; Grospretre et al. 2016), but little is known about the neural mechanisms underlying the effects of AO + MI. There is evidence that the movement-related corticospinal excitability is higher during AO + MI than during AO or MI alone (Ohno et al. 2011; Sakamoto et al. 2009; Tsukazaki et al. 2012; Wright et al. 2014, 2016). Furthermore, we showed task-specific modulation of corticospinal excitability during AO + MI, with greater facilitation during a complex compared to an easy postural task (Mouthon et al. 2015, 2016). Similarly, imaging studies revealed greater brain activity (in motor centers) during AO + MI compared to AO or MI that was task-specifically modulated (for review see Eaves et al. 2016b; Vogt et al. 2013). Interestingly, some studies even demonstrated that the brain activity in motor centers during AO + MI was greater than the sum of the activity during AO and MI alone (e.g., Sakamoto et al. 2009; Taube et al. 2015).

One aspect of AO + MI, which has not been investigated so far, is the implication and modulation of the inhibitory system. For MI and AO, intracortical inhibition was shown to be decreased when imagining or observing motor actions compared to a resting control condition (Abbruzzese et al. 1999; Battaglia et al. 2011; Patuzzo et al. 2003). In addition, intracortical inhibition was adapted in relation to the muscle and the temporal characteristics of the movement during MI (Stinear and Byblow 2004).

Therefore, the first aim of the current study was to elucidate whether changes in intracortical inhibition during the mental simulation of postural tasks were comparable to the changes observed when the same tasks were physically executed. The second aim was to investigate inhibitory processes within different forms of mental simulation. The study also had a look at the modulation of corticospinal excitability and background EMG activity (bEMG). For this purpose, modulation of intracortical inhibition, corticospinal excitability and bEMG during AO + MI were compared to the modulation with AO alone.

In a first step, a paired pulse transcranial magnetic stimulation (TMS) paradigm was used to assess short-interval intracortical inhibition (SICI) during physical execution and mental simulation of two postural tasks: a stable and an unstable standing task. In a second step, we compared SICI during AO + MI with SICI during AO alone to reveal differences in intracortical inhibition for different forms of mental simulation. We decided to compare AO + MI with passive AO but not with MI as this allows a better (temporal) control of the experiment as participants had to follow a video sequence, which is not possible with MI. Thus, AO + MI and AO both have the advantage that the internal representation of the observed motor action is timely synchronized with the corresponding movement. Furthermore, previous studies reported more pronounced differences between AO + MI with AO alone than MI alone (Mouthon et al. 2015; Sun et al. 2016; Taube et al., 2015; 2015). Finally, changes in the corticospinal excitability using single TMS pulses, and bEMG were also assessed to get a better picture of the neural processing involved in AO + MI.

Based on the above cited literature, we hypothesized that switching from the stable to the unstable stance condition would result in decreased intracortical inhibition and increased corticospinal excitability during physical task execution, and modulation during mental simulation conditions would mirror the modulation observed in the executive condition with stronger effects for AO + MI than for AO, whereas bEMG should remain largely unchanged in the mental simulation conditions.

## Methods

### Participants

Twenty-one young adults (mean age ± SD = 24 ± 6.3 years; 5 females) were integrated into the final analysis of this study. The required number of participating subjects was estimated based on an a priori power analyses based on Faul et al. (2007) with the following assumptions: effect size 0.25, alpha 0.05, power 95%, repeated measures of ANOVA. The power analyses revealed that 25 subjects needed to be included in the study. 26 participants performed the study, but only 21 were included in the final analysis as in 4 of them, we could not obtain all data from all conditions. Inclusion criteria for this study were to be between 18 and 35 years and to be in good health condition. Participants presented the following conditions were excluded; people who have/had severe orthopedic disorders, epilepsy crisis or previous family history, implant as prosthesis or cardiac simulator, splinters of metal inside the body, suffered cerebral stroke or neurology disorders, severe head trauma, heart problem, people who takes drugs. All participants gave their written consent to the experiment, which was approved by the local Ethics Committee.

### Experimental protocol

**Fig. 1:**
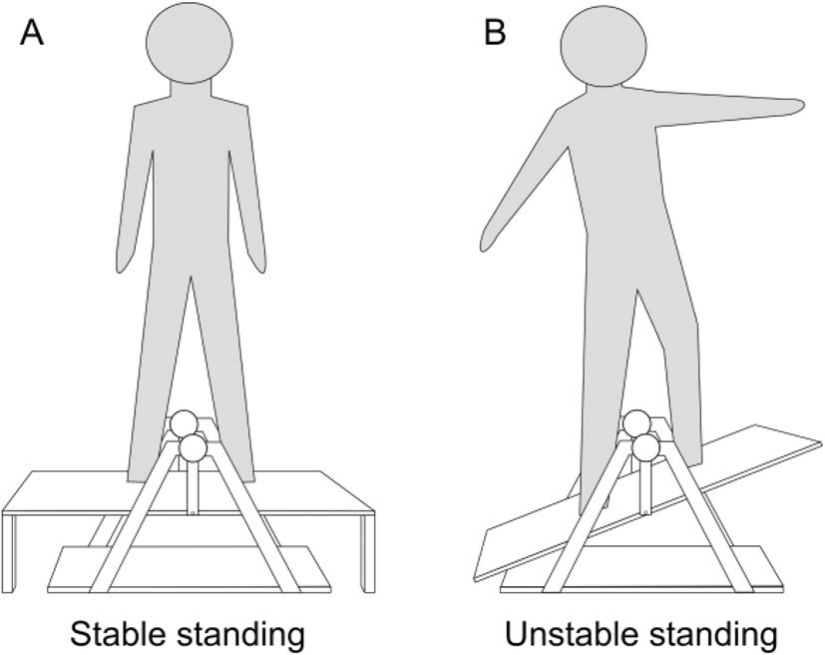
Postural tasks performed during the experiment. **a** Standing on stable ground (stable standing). **b** Standing task on a stability platform that is freely moving (unstable standing). For the mental simulation conditions, the participants watched videos of a person performing the two tasks

Corticospinal excitability and intracortical inhibition were assessed during two standing tasks of different difficulty. Both tasks consisted on standing on a stability platform (Model 16,030, Lafayette Instrument Company®, USA). bEMG was also recorded to observe changes in muscular activity during physical and mental performance of the balance tasks. This helped to consider whether changes in SICI and MEP were due to changes at a spinal or a supraspinal level. In the first condition, the platform was stable (stable standing), in the second condition, it was freely moving in lateral directions (unstable standing; see Fig. 1).

Both tasks were performed physically and mentally. For the mental simulation, the participants—lying in a supine position—watched videos of a person performing the two tasks. Each participant simulated the two tasks using two techniques: (1) passive AO and (2) AO + MI. For AO, the instruction was to “passively” watch the video without further mental effort. For AO + MI, the participants were instructed to watch the video and, at the same time, imagine that they were executing the task themselves. The participants were introduced to the tasks and familiarized with the videos by the experimenter just before the experiment.

### EMG recording

Motor evoked potentials (MEPs) evoked by TMS (see next section) were recorded from the right soleus muscle (SOL) by means of electromyography (EMG). For that purpose, bipolar surface electrodes (Blue sensor P, Ambu®, Bad Nauheim, Germany) were placed over the SOL. The reference electrode was attached on the tibia plateau. The EMG signals were amplified (1000x), sampled at 4 kHz, and band-pass filtered (10–1000 Hz). Data were recorded using custom-made software (LabView®-based, National Instruments®, Austin, Texas).

### TMS protocols

TMS was used to quantify corticospinal excitability and short-interval intracortical inhibition (SICI). Single and paired TMS pulses were applied using a 95-mm “butterfly-shaped” coil (D-B80) and a MagPro X100 with MagOption magnetic stimulator (both MagVenture A/S, Farum, Denmark). MEPs were elicited in the right SOL by stimulating over the left M1. At the beginning of the session, the motor hot spot of the SOL was determined by shifting the coil until the optimal position for eliciting MEPs was found with low stimulation intensity. The location was marked on the skull to check whether the coil moved during the experiment. During the physical conditions, the coil was fixed to the participant’s head with a custom-built helmet (Ruffieux et al. 2017). During the mental conditions, it was fixated using a tripod.

In a next step, the active motor thresholds (aMTs) for the executive conditions were determined for the stable and unstable task to reduce the MEP variability between the physical conditions, whereas only one threshold, i.e., the resting motor threshold (rMT), was used for all the mental conditions. In other words, three motor thresholds were determined, one for the stable task of the executive condition, one for the unstable task of the execution conditions and one (resting threshold) for all mental conditions. The aMTs and rMT were determined as the lowest stimulation intensity that elicited an MEP higher than 100 µV in three out of five trials (Kujirai et al. 1993b).

In each condition, single and paired pulses were applied to assess corticospinal excitability and SICI, respectively. SICI was elicited using a paired pulse paradigm that consisted of applying a subthreshold conditioning stimulus that activates intracortical interneurons which alter the excitability of corticospinal neurons and thus, modulate the response to a subsequent suprathreshold test stimulus (Chen et al. 1998; Kujirai et al. 1993a). When short interstimulus intervals are used (1–5 ms), the MEPs produced by the test stimulus present smaller amplitudes (i.e., inhibition). This is in all likelihood due to the activation of GABA_A_-ergic inhibitory interneurons (Ilic et al. 2002; Ziemann et al. 1996). For the paired pulses, the stimulation intensities for the conditioning and the test pulses were set to 80 and 120%, respectively, of the respective MTs (active or resting), and we used an inter-stimulus interval of 2.5 ms based on the literature (Papegaaij et al. 2014; Roshan et al. 2003). The stimulation intensity for the single pulses (control MEPs) was set to 120% of the respective MTs.

During each physical condition (stable and unstable standing), 20 single and 20 paired pulses were applied in an alternating order, with an inter-stimulus interval of 4 s.

During each of the four mental conditions (2 simulation techniques × 2 tasks), six single pulses and six paired pulses were applied in an alternating order. Before and after each condition, a resting control condition of 24 s was included in which participants were instructed to look at a cross on the screen. This procedure was repeated twice to control for fatigue or changes in attention. Thus, 12 control and 12 conditioned MEPs were recorded in each mental simulation condition across the whole experiment. The inter-stimulus interval was set to 4 s. The four conditions were displayed in a random order.

### Data analyses

To quantify corticospinal excitability and SICI, we computed the peak-to-peak amplitudes of the conditioned and control MEPs. SICI was expressed as percentage of inhibition using the following formula: 100—(conditioned MEP/test MEP × 100) (Kuhn et al. 2013, 2017; Papegaaij et al. 2014).

To assess the influence of the bEMG during the execution and mental simulation of the tasks, the root mean square of the bEMG signal was calculated for a time interval of 100 ms before the stimulation and reported as absolute values.

### Statistical analyses

First, the SICI was analysed by comparing the change in SICI between executed and mentally simulated balance tasks with a two-way repeated measures ANOVA with the factors CONDITION (Execution vs. AO + MI vs. AO), and BALANCE TASK (unstable vs. stable).

Secondly, changes in SICI from the stable to the unstable task were compared across conditions with a one-way repeated measures ANOVA with the factor CONDITION (Execution vs. AO + MI vs. AO) to detect potential differences between conditions.

Finally, to explore the neural processing of mental simulation and to contrast AO + MI and AO, we performed a two-way repeated measures ANOVA with the factors CONDITION (AO + MI vs. AO) and BALANCE TASK (unstable vs. stable) for SICI of the two mental simulation conditions.

Changes in corticospinal excitability were analysed in two different ways. First, to compare differences between the three conditions (Mental simulation vs. Execution stable task vs. Execution unstable task), we performed an indirect comparison of changes in corticospinal excitability by comparing the MTs used for the execution of the stable and unstable task and the mental simulation (i.e., RMT). MTs were put in a one-way repeated measures ANOVA with the factor CONDITION (Execution vs. AO + MI vs. AO). This analysis was made, because the setup of the study did not allow a direct comparison of the corticospinal excitability between the three conditions as the MT was adapted in the execution condition between the stable and unstable standing, but not between the stable and unstable condition of the mental simulation procedure.

Secondly, we used MEP amplitudes to explore the neural processing of mental simulation and to contrast AO + MI and AO as the RMT used was the same between both balance tasks, and a direct comparison of the corticospinal excitability was possible. Analysis of MEP amplitudes was performed using a two-way repeated measures ANOVA with the factors CONDITION (AO + MI vs. AO), and BALANCE TASK (unstable vs. stable).

Investigation of changes in bEMG activity was performed with a two-way repeated measures ANOVA with the factors CONDITION (Execution vs. AO + MI vs. AO), and BALANCE TASK (unstable vs. stable) was used. Besides, a Pearson correlation analysis was performed between changes in SICI and bEMG activity for the execution condition to estimate the impact of the bEMG on SICI.

A Greenhouse–Geisser correction was performed for every ANOVA when the assumption of sphericity was violated. All significant main effects from ANOVA statistic were followed up by post hoc Student’s *t* tests with Bonferroni correction. Data are displayed as mean ± standard deviation (SD). The size effects were calculated for ANOVA using Generalized Eta-Squared measure (Bakeman 2005) and Student’s *t* tests using Cohen’s *d* (Cohen 1988). The significance level was determined at *p* < 0.05. All statistical analyses were calculated with the software *R* (R Core Team 2013).

## Results

### SICI results

#### Execution vs. AO + MI vs. AO

**Fig. 2:**
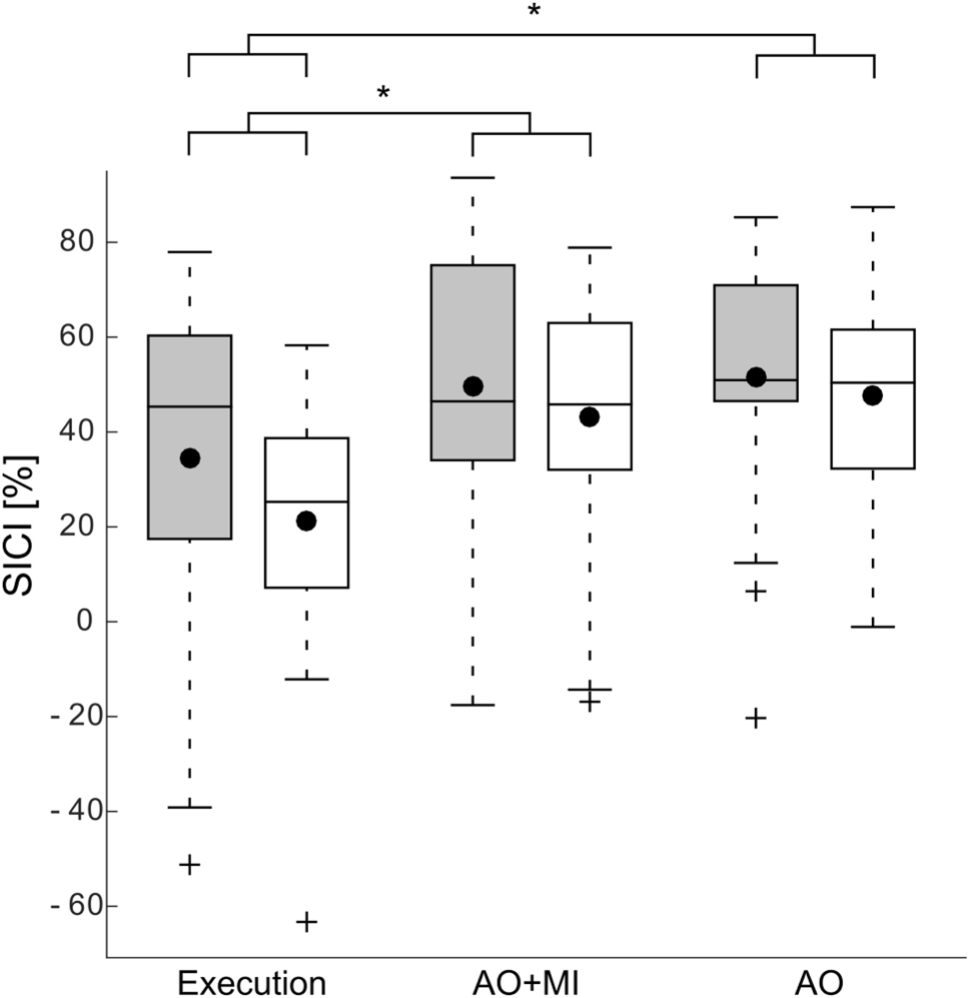
Changes in SICI during execution and mental simulation of balance tasks for the soleus muscle. SICI displayed a significant decrease when participants switched from the stable to the unstable standing task during both execution and mental simulation of postural tasks (*F*_1,20_ = 8.34, *p* = 0.009, *η*^2^ = 0.02). During the execution of balance tasks, intracortical inhibition was lower than during the mental simulation conditions AO + MI (*t* = 2.6; *p* = 0.009; *d* = 0.6) and AO (*t* = 2.7; *p* = 0.002; *d* = 0.6). Gray and white bars represent stable and unstable standing tasks, respectively. The black dots represent the mean values, while the horizontal lines within the boxes indicate the median values. The box covers the 25th–75th percentiles, the whiskers represent the range, and the black crosses indicate outliers (*p < 0.05)

Analysis of SICI between conditions (i.e., execution, AO + MI and AO) and balance tasks (i.e., stable vs. unstable) revealed main effects for CONDITION (*F*_2, 40_ = 6.42, *p* = 0.009, *η*^2^ = 0.1) and BALANCE TASK, (*F*_1,20_ = 8.34, *p* = 0.009, *η*^2^ = 0.02), but no significant interaction (see Fig. 2; data presented in Tables 1 and 2). This means that there is a decrease of SICI when participants switch (physically or mentally) from the stable standing task to the unstable task. In addition, post-hoc tests confirmed less intracortical inhibition in the execution condition compared to the AO + MI (*t* = 2.6; *p* = 0.009; *d* = 0.6) and AO (*t* = 2.7; *p* = 0.002; *d* = 0.6) condition. No significant difference was found between AO + MI and AO (*t* = -0.7; *p* = 0.9; *d* = 0.2).

**Table 1:** Mean ± sd of SICI, MEP amplitudes and bEMG activity for the three conditions; execution, action observation during motor imagery (AO + MI) and action observation (AO)

	Execution	AO + MI	AO
SICI [%]	27.8 ± 32.9	46.36 ± 27.3	49.5 ± 20.3
MEP [mV]	0.91 ± 0.5	0.19 ± 0.11	0.15 ± 0.08
bEMG [mV]	0.052 ± 0.03	0.003 ± 0.003	0.002 ± 0.002

**Table 2:** Mean ± sd of SICI, MEP amplitudes and bEMG activity for the stable and unstable balance tasks in all conditions; execution, action observation during motor imagery (AO + MI) and action observation (AO)

	Execution		AO + MI		AO	
	Stable	Unstable	Stable	Unstable	Stable	Unstable
SICI [%]	34.4% ± 34.4	21.2% ± 27.4	49.5% ± 27.6	43.2% ± 27.4	51.5% ± 26.6	47.54% ± 23.5
MEP [mV]	0.92 ± 0.6	0.90 ± 0.2	0.19 ± 0.09	0.21 ± 0.1	0.16 ± 0.08	0.15 ± 0.08
bEMG [mV]	0.042 ± 0.02	0.062 ± 0.04	0.0030 ± 0.003	0.0034 ± 0.003	0.003 ± 0.003	0.002 ± 0.001

The one-way ANOVA between difference of SICI between the stable and unstable for all conditions was not significant (*F*_2, 40_ = 0.6, *p* = 0.5, *η*^2^ = 0.02).

#### AO + MI vs. AO

SICI recorded during the mental simulation conditions showed a main effect of BALANCE TASK (*F*_1,20_ = 20.1; *p* < 0.001; *η*^2^ = 0.01) with a reduction of 10% in the unstable balance task compared to the stable task. Neither the main effect of CONDITION was significant (*F*_1,20_ = 0.3; *p* = 0.6; *η*^2^ = 0.004) nor the interaction of CONDITION x BALANCE TASK (*F*_1,20_ = 0.1; *p* = 0.7; *η*^2^ < 0.001; data presented in Tables 1 and 2).

### Motor threshold and MEP results

#### Mental simulation vs. Execution of stable and unstable tasks

**Fig. 3:**
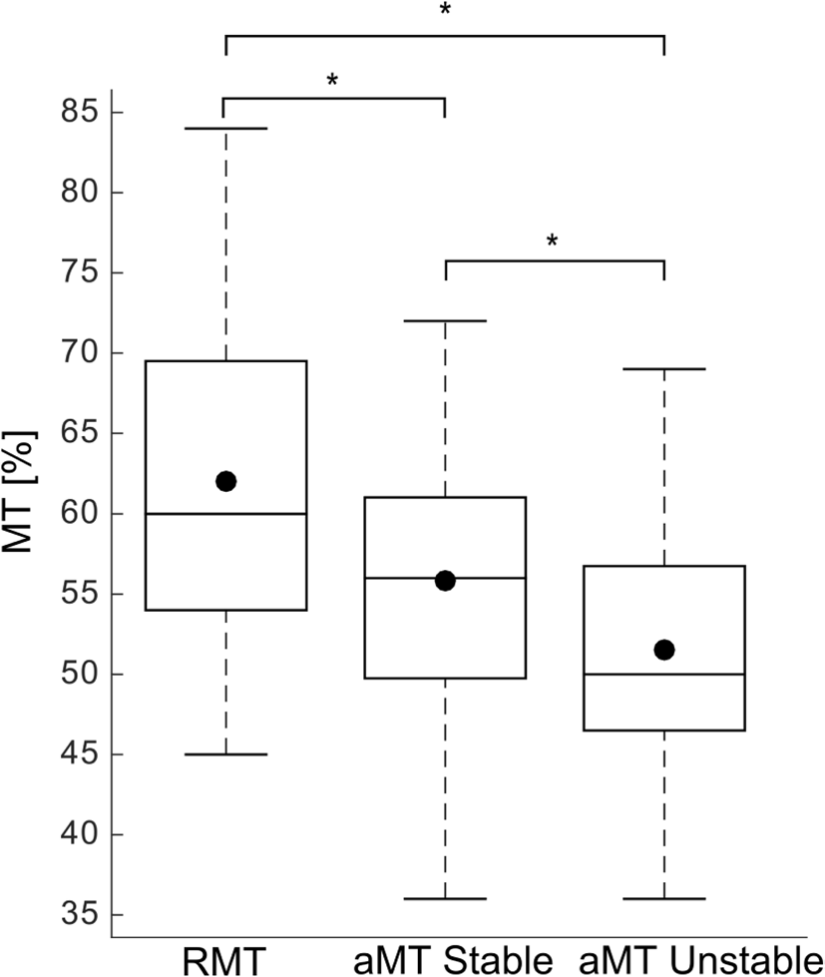
Results of the motor threshold (MT) used during execution and mental simulation. The figure displays the resting motor threshold (RMT) used for the two mental simulation tasks (AO and AO + MI; 62.1% ± 2.3) and the active motor thresholds (aMTs) that were applied for the execution of the stable (55.9% ± 1.9) and unstable balance tasks (51.6% ± 1.8). The black dots represent the mean values, while the horizontal lines within the boxes indicate the median values. The box covers the 25th–75th percentiles, the whiskers represent the range, and the black crosses indicate outliers (*p < 0.05)

Comparison of the MTs used in the three conditions, as the setup of the study did not allow a direct comparison of the corticospinal excitability between the conditions, showed significant differences between conditions with a main effect of CONDITION (*F*_2_, _40_ = 28.2, *p* < 0.001, *η*^2^ = 0.02; see Fig. 3). Post-hoc tests confirmed a significant difference between the RMT of mental simulation and aMT of the stable task (*t* = 5.3; *p* < 0.001; *d* = 1.2), and unstable task (*t* = 5.8; *p* < 0.001;* d* = 1.3), as well as between aMT of the stable and unstable task (*t* = 3.7; *p* = 0.003; *d* = 0.8).

#### AO + MI vs. AO

**Fig. 4:**
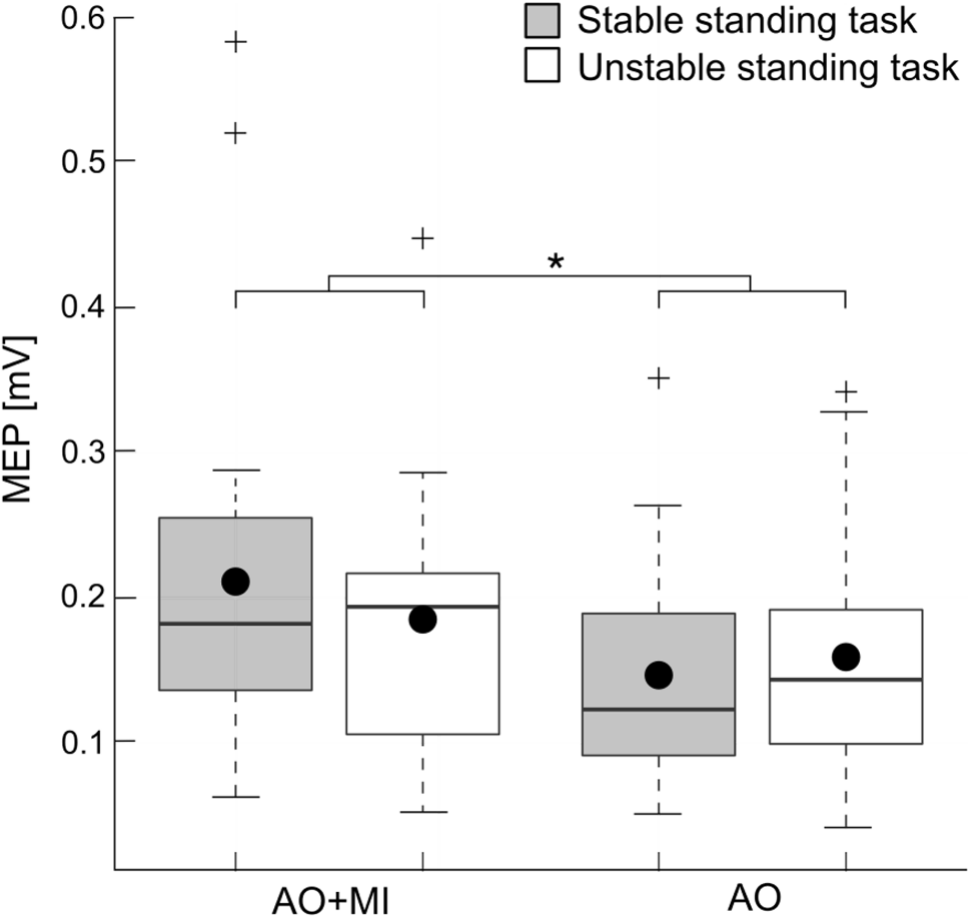
Changes in MEP amplitudes during mental simulation of balance tasks for the soleus muscle. Significant changes were found in the corticospinal excitability between action observation during motor imagery (AO + MI) and ‘passive’ action observation (AO; *F*_1,20_ = 20.5; *p* < 0.001, *η*^2^ = 0.05). Gray and white bars represent stable and unstable standing tasks, respectively. The black dots represent the mean values, while the horizontal lines within the boxes indicate the median values. The box covers the 25th–75th percentiles, the whiskers represent the range, and the black crosses indicate outliers (*p < 0.05)

A direct comparison of MEP amplitudes between AO + MI with AO revealed that MEP amplitudes were influenced by the factor CONDITION (F_1,20_ = 20.5; *p* < 0.001, *η*^2^ = 0.05) with a 29.5% larger effect for AO + MI compared to AO (see Fig. 4; data presented in Tables 1 and 2). There was neither a main effect of BALANCE TASK (*F*_1,20_ = 0.1; *p* = 0.7, *η*^2^ = 0.001) nor a significant interaction CONDITION x BALANCE TASK (*F*_1,20_ = 3.2; *p* = 0.08, *η*^2^ = 0.009).

### Background EMG activity

**Fig. 5:**
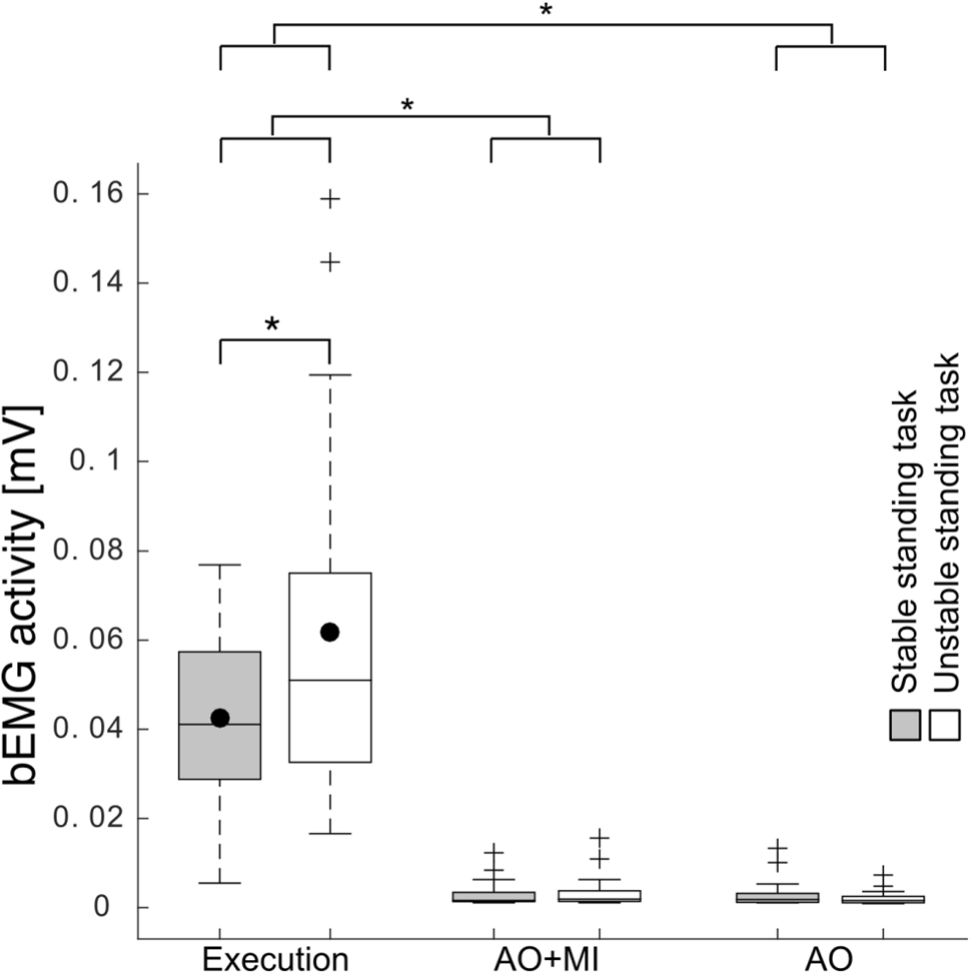
bEMG activity during execution and mental simulation of the stable (gray bars) and unstable (white bars) balance task for the soleus (SOL) muscle. Significant changes in the bEMG activity between the stable und unstable standing task were only found during physical execution of the tasks (*p* = 0.03) but not during mental simulation. The switch from the execution condition and mental conditions reduced significantly the bEMG (Execution vs. AO + MI; *p* < 0.001 and Execution vs. AO; *p* < 0.001), but the change of bEMG activity between AO + MI and AO was not significant (*p* = 1). The black dots represent the mean values, while the horizontal lines within the boxes indicate the median values. The box covers the 25th–75th percentiles, the whiskers represent the range, and the black crosses indicate outliers (*p < 0.05)

Overall, analysis of the bEMG revealed significant main effects of CONDITION (*F*_2,40_ = 76.5; *p* < 0.001, *η*^2^ = 0.6) and BALANCE TASK (*F*_1,20_ = 5.7; *p* = 0.02, *η*^2^ = 0.03) as well as a significant interaction of CONDITION x BALANCE TASK (*F*_2,40_ = 5.8; *p* = 0.006, *η*^2^ = 0.05; see Fig. 5; data presented in Tables 1 and 2).

Unsurprisingly, post hoc tests revealed that the switch from a standing (execution) to a lying position (mental conditions) significantly reduced the bEMG. Thus, there were large differences between the physically executed and the mentally performed tasks (Execution vs. AO + MI; *t* = − 8.8; *p* < 0.001; *d* = 1.9, and Execution vs. AO; *t* = − 8.7; *p* < 0.001; *d* = 1.9). However, the difference of bEMG activity between AO + MI and AO was not significant (*t* = 1.3; *p* = 1; *d* = 0.3).

The comparison between the stable and the unstable balance task for each condition (Execution, AO + MI, and AO) showed significantly larger activity in the unstable standing condition compared to the stable standing condition for the physically performed task (Execution: *t* = − 2.4; *p* = 0.03; *d* = 0.5; Fig. 5), but not for the AO + MI (*t* = − 0.9; *p* = 0.7; *d* = 0.2) and AO (*t* = 1.2; *p* = 0.4; *d* = 0.3; see Fig. 5) conditions.

However, for the physically executed tasks, no significant correlation was found between the changes in SICI and changes in bEMG activity when switching from the stable to the unstable standing (*r* = − 0.3; *p* = 0.2).

## Discussion

To our knowledge, this is the first study comparing task-specific adaptations of intracortical inhibition during physically performed balance tasks with the changes in SICI during mental simulation of the same tasks. Our results revealed that intracortical inhibition was similarly modulated during execution and mental simulation of balance tasks. In both cases, lower SICI was observed in the unstable standing task than in the stable task with a greater difference during execution (− 38.5%) than during mental simulation (AO + MI − 12.8%; AO − 7.6%). However, these differences were not statistically different indicating that the modulation was similar between all conditions.

### Task-specific adaptation of intracortical inhibition during physical execution

Our results revealed that executing a challenging balance task compared to a simple balance task reduced SICI. This result is well in line with previous studies consistently showing a decrease of inhibition with increased postual task difficulty (Papegaaij et al. 2014, 2016a, b; Soto et al. 2006). Some authors argued that this decrease might help to increase the readiness state of M1 so that it becomes more easily activated in the case maintenance of posture is threatened (Papegaaij et al. 2016a). In the same way, the decrease of the active motor threshold during the more challenging postural task revealed indirectly an increase of the corticospinal excitability, and may further support this argument. Alternatively, the reduction of SICI may ensure the activation of the muscles involved in the control of the ankle and thus, influence ankle stiffness. Nonetheless, this seems improbable, as the modulation of SICI was not related to changes in bEMG activity. Indeed, no correlation was found between changes in bEMG activity and SICI despite the fact that during execution, bEMG was significantly higher in the more challenging balance task. Moreover, the aMT was determined for the stable and unstable standing task and stimulation intensity was consequently adapted to decrease influence of changes of the bEMG on the SICI results. This resulted in comparable MEP amplitudes in the executed stable and unstable task as can be seen in Table 2. Thus, the modulation in SICI was most probably due to the increase in postural task difficulty and not a consequence of changes in the bEMG activity.

### Similarities between execution and mental simulation of balance tasks

In line with our observation during physical task execution, a task-dependent change in SICI during mental simulation of balance tasks (i.e., AO + MI and AO taken together) was observed resulting in a decreased SICI in the unstable compared to the stable task. For the first time, this finding highlights that SICI is similarly modulated with respect to postural task difficulty during physical task execution and during mental simulation of balance tasks. It has to be emphasized that not many studies have indeed compared real task execution with mental simulation of the exact same task in the same subjects. For balance control, this is actually the first one; at least to our knowledge. This comparison reveals that during physical task execution inhibition is less pronounced than during mental simulation. More importantly, in all conditions a decrease in SICI was observed when participants switched from the stable to the unstable standing task. The lack of interaction between condition and balance task indicates a similar modulation from stable to unstable between physical and mental conditions. In this way, our results strengthen the assumption that there is a considerable overlap and similarity between executed and mentally simulated actions and, therefore, support Jeannerod’s hypothesis that “the motor system is part of a simulation network that is activated under a variety of conditions in relation to action, either self-intended or observed from other individuals” (Jeannerod 2001). This simulation network was shown to be specifically activated by different covert actions such as MI, AO or AO + MI although Jeannerod assumed a core network that pertains to all stimulation states (Jeannerod 2001). When taking into account the most recent findings about covert actions, there is growing evidence that AO + MI seems to be the most effective way to activate sensorimotor centers and may, therefore, be the closest resemblance to real task execution (Eaves et al. 2016b; Vogt et al. 2013). This was also shown for balance tasks (Mouthon et al. 2015; 2016;2018; Taube et al. 2015). The current results only partly support this assumption. Unlike our initial hypothesis that changes in SICI would be stronger in AO + MI than in AO, our results demonstrated that the type of mental simulation did neither impact the amount nor the modulation of intracortical inhibition. However, analysis of the corticospinal excitability during mental simulation revealed a larger increase of the MEP facilitation in the AO + MI condition compared to the AO condition, which is in line with previous studies (Mouthon et al. 2015; Mouthon and Taube 2019).

Finally, the current results extend previous suggestions about the promising role of AO + MI as a rehabilitation tool (see Eaves et al. 2016b, a) and underline the high potential of AO + MI for rehabilitation of postural control. Indeed, the direct and indirect comparison of AO + MI with motor execution aimed to show that the neural mechanisms of AO + MI are closer to the mechanisms of real task execution compared to AO, and thus more efficient for non-physical rehabilitation and motor learning. However, the current study does not provide evidence that AO + MI is more closely linked to a real execution condition compared to MI. Therefore, further research should be conducted to (1) directly compare the impact of AO + MI, MI and an execution condition, (2) assess changes in brain activity and motor cortical excitability in response to non-physical balance training to better understand the effects of AO + MI on the neural processing of mental simulation and postural control, (3) better determine which population would benefit the most from this form of intervention, and (4) how best to deliver AO + MI to participants.

## Conclusion

Intracortical inhibition was task-dependently modulated during physical balance execution and mental simulation of the same balance tasks. More specifically, increased postural task difficulty induced a reduction of SICI during execution (− 38.5%) and during mental simulation (AO + MI − 12.8%; AO − 7.8%). Therefore, we conclude that the neural processing of mental simulation of balance tasks shares high similarities with the processing of physical balance tasks, comprising intracortical inhibitory processes.

## Abbreviations

AO: Action observation
aMT: Active motor threshold
ANOVA: Analysis of variance
bEMG: Background EMG
EMG: Electromyography
MEPs: Motor evoked potentials
MI: Motor imagery
AO + MI: Motor imagery during action observation
MT: Motor threshold
M1: Primary motor cortex
rMT: Rest motor threshold
SICI: Short-interval intracortical inhibition
SD: Standard deviation
SOL: Soleus muscle
TMS: Transcranial magnetic stimulation
